# Automatic Extraction of Power Cables Location in Railways Using Surface LiDAR Systems

**DOI:** 10.3390/s20216222

**Published:** 2020-10-31

**Authors:** Alexis Gutiérrez-Fernández, Camino Fernández-Llamas, Vicente Matellán-Olivera, Adrián Suárez-González

**Affiliations:** 1 Módulo de Investigación en Cibernética, University of León, Campus de Vegazana S/N, 24071 León, Spain; camino.fernandez@unileon.es (C.F.-L.); vicente.matellan@unileon.es (V.M.-O.); 2 Teléfonos, Líneas y Centrales S.A. (Telice S.A.), Polígono Industrial de Onzonilla Fase-II, Calle C, Parcela M-24, 24391 Ribaseca, Spain; asuarez.gonzalez@telice.es

**Keywords:** railway, overhead contact line, LiDAR, automatic point cloud analysis

## Abstract

The assembly and maintenance of electrified railway systems is of vital importance for its correct operation. Contact wires are critical elements since the correct collection of energy from trains through pantographs depends on them. Periodical inspection of the state of these installations is essential. This task traditionally implies a heavy manual workload subject to errors. A new system that allows one to check the state (height and stagger) of contact and messenger wires is presented on this article blueA new method based on seven steps for identifying the contact wires and measuring their height and stagger from point clouds recorded by means of a LiDAR system is presented. This system can be used both in assembly and maintenance phases, as well as afterwards, allowing the analysis of point clouds previously recorded. The new method was evaluated in both test bench and real environments against the commonly used measurement method. Results with the comparison between this new system and the commonly used measurement method in both test bench and real railway environments are presented. Results of this comparison show differences of less than a centimetre on average and the amount of time spent for the measuring phase is significantly decreased and not prone to human errors.

## 1. Introduction

The railway has become one of the most important means of transport around the world in recent years, not only for passengers but also for goods. Governments from different countries are developing sustainable mobility strategies in which the railway will play a leading role against environment and climate change challenges [1], caused by its low carbon footprint compared to other transports.

The development of increasingly efficient railway electrification systems is mandatory in this scenario, with the focus on the increasingly widespread high-speed lines that join existing railway environments such as urban and interurban corridors or conventional railways. It is necessary to carry out analysis and maintenance tasks on these infrastructures to preserve them in good conditions for a safe and reliable operation.

Contact wires are critical elements in this kind of electrified systems because their geometric position with respect to the rail level and to the track axis (height and stagger) are closely related with a good energy collection through the pantograph of the train. Lower maintenance costs can be achieved if contact wires are well positioned because less wear is produced due to friction. Therefore, it is necessary to carry out regular inspections (both in assembly and operation phases) looking for deviations between the as-built infrastructure and the analysed one in order to ensure a good state and position of the contact wires in the electrified railway infrastructure.

**Figure 1:**
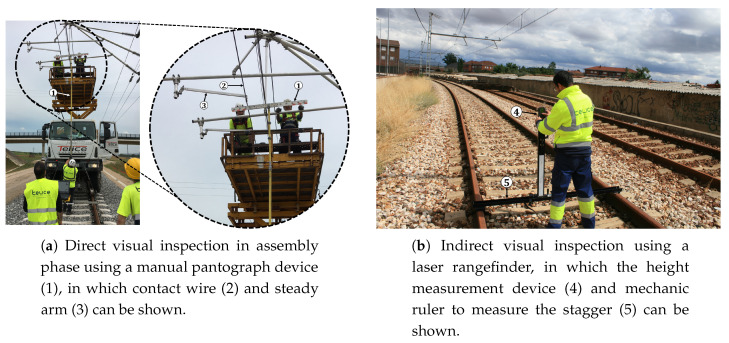
Direct and indirect visual inspection.

The maintenance task is normally performed by workers using manual inspection methods, both direct inspection (in assembly phase, as shown in Figure 1a) and indirect inspection (with laser rangefinders, as shown in Figure 1b). These are slow and low productive processes that involve a heavy workload prone to errors (visual readings, take notes on the field, manual data entry in the system, etc.), so its reliability is highly dependent on the operator knowledge and skills. However, the advancement of technology in matters related to capturing the environment and object recognition has increased the possibilities to automate the inspection of this type of environment with manual operated equipment.

Inspection trains are the alternative for monitoring overhead contact lines and they include several measurement devices. However, the acquisition of these vehicles implies large purchases and operating costs (i.e., 7.83M € for BT 355.001 and BT 355.002 models and more than 43M € for “Doctor Avril” model, specifically designed for inspecting high speed corridors in Spain. The operation costs for five years are included in both cases). They are only functional in long sections (complete line or corridor) and within the framework of maintenance planned by the manager of the infrastructure (often once or twice a year). In addition, the huge amount of data generated makes its management hard. It is common to generate reports where the defects detected in the geometric inspection are summarised and classified in severity levels according to predefined thresholds. Based on these defects of each geometric parameter, generic quality indexes are defined that allow planning the subsequent interventions that are necessary. Therefore, the scope of these systems is limited, making it unsuitable for the daily quality control of a project or as a diagnostic tool for urgent and unexpected problems, fields in which manually operated equipment that makes use of new measurement technologies are clearly better positioned.

LiDAR technology (Laser imaging Detection And Ranging) is positioned as one of the most widely used technologies for capturing data in this kind of environment, since it allows capturing large amounts of data in a short period of time. However, the quality of the data obtained by LiDAR sensors varies depending on their capabilities and on the systems that complement them, which together allow us to capture complete information about the environment.

Commercial LiDAR devices [2] generally have a lower measurement speed and accuracy than systems specifically designed and used in railway environments. A cost reduction in the development of measurement systems for railway environments motivates the use of this kind of sensors.

**Figure 2:**
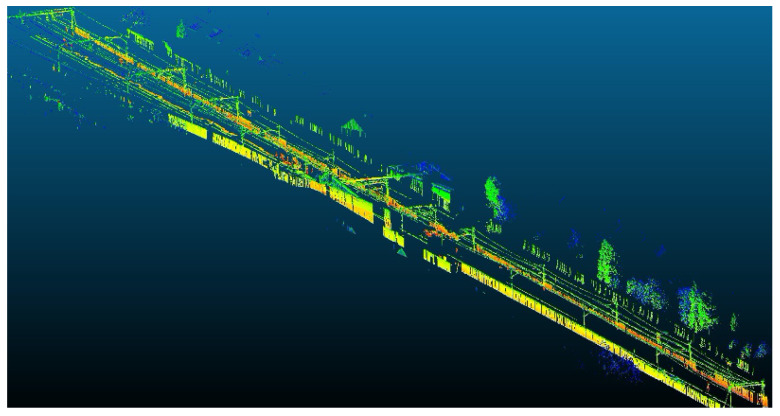
Point cloud provided by the Telice company of a track with standard gauge and flexible overhead contact line.

A point cloud recorded by a 2D commercial LiDAR from Telice company is shown in Figure 2. This point cloud has been recorded in London and it belongs to a standard gauge (1435 mm) railway section, electrified by means of a flexible overhead contact line system.

In this context, the goal of this work is the development of a new system capable of checking both contact and messenger wires status (height and stagger) in a railway infrastructure. The final system where this new method will be integrated should include the following characteristics: (1) suitable for both assembly and maintenance phases; (2) compatible with commercial LiDAR systems; (3) portable structure to be carried to the rail without the need of a train to be used; (4) save time over actual methods performed manually while offering the operators real time measures; (5) higher level of automation to avoid human errors.

An analysis about studies in which LiDAR sensors have been used to record and to identify elements in the environment is performed in Section 2. Section 3 details the new method for automatic recognition and measurement of contact wires on commercial LiDAR recorded point clouds. In Section 4 the results of applying the new method to several data sets are shown and in Section 5 a discussion about these results is exposed. Finally, in Section 6 both conclusion and future lines of work are presented.

## 2. Related Work

LiDAR technology is used in a variety of systems in which obtaining data in a quick and accurate way is needed. Besides, this technology allows us to obtain these data avoiding physical contact with the scanned infrastructures [3]. Zhang et al. [4] have presented a curb detection system based on the data captured by a LiDAR and that is applied to autonomous driving. There are also several studies that have worked with point clouds collected with LIDAR sensors from railway environments. Most of these point clouds have been captured by a Mobile LiDAR System (MLS), which consists of capture units and navigation systems for their positioning in space, and which allow data to be captured quickly, safely and with a high level of quality and accuracy [5]. The most basic studies with this kind of systems are limited to data collection and their further visual inspection with the goal of identifying defects in the infrastructure, such as the one from Morgan [6].

Other studies have performed automated tasks on point clouds acquired by an MLS from a railway environment. Che et al. [7] have presented a systematic review focused on identifying studies in which object recognition methods based on data recorded by LiDAR sensors in different scenarios are developed. In a railway environment, most of the studies are focused on identifying the track bed and the rails from the point cloud [8,9,10,11,12].

On the other hand, there are studies in which point clouds are processed in order to identify elements beyond the rails and the track bed. In Arastounia’s work [13], air-collected point clouds from systems mounted in drones or light planes are used and specific analysis methods for the characteristics of this kind of data sets are applied. In this work, the identification of the characteristic elements in the infrastructure is based on a first phase of seed detection for each element and a second phase of growth that allows the detection of all the points that make up an element from its seed. However, the inferior quality of the air-collected data implies the need to carry out the seed selection phase manually.

The studies carried out with point clouds taken from MLS ground systems have a very high quality and density of points, allowing automated techniques to be applied in each of the detection phases. Yoon, J.S. et al. [14] propose a method based on the study of points’ intensities that allows for the identification of the ones that belong to the railway infrastructure in a tunnel point cloud and to detect if there is any damaged area. In the study performed by Sanchez-Rodríguez et al. [15] and extended in [16], tunnel point clouds are also processed in order to classify the elements that appear in them. Specifically, those points that belong to the overhead contact line are identified applying the RANSAC adjustment method [17]. Arastounia, in his previously cited work [13], applies two different methods in order to classify the points: a template matching based method and a region growing based method. In the first one (template matching), a preliminary points classification is refined based on the morphology that such elements are expected to have in the infrastructure (i.e., it is expected that contact wire lies between rails at a concrete height range). In the second method, the preliminary classification is used to define a seed of each element and then a grow algorithm is applied that allows adding to the wire those points in the cloud that are actually part of it.

Arastounia’s successive studies have improved the identification process of elements in a railway infrastructure and, more specifically, the detection of its contact wires. In one of them [18], a Principal Component Analysis (PCA) algorithm is applied to some sections of the point cloud to identify the elements that produce the greatest variation in the eigen values (straight lines), allowing the detection of contact wires. In another study [19], an improved grow algorithm is applied in parallel between the rails and the contact wires, which allows the simultaneous detection of both elements.

There are also some documents in the patent literature that define procedures for measuring geometric parameters of the contact wire based on LiDAR data. H. Liping [20] and C. Youqi et al. [21] have presented patents applications for mobile height and stagger measurement systems for overhead lines, as well as its associated measurement methods. This methodology is based on simple filtering, fusion and statistical treatment—linear regression—processes of the lowest points found above the sensor, all of them in real time. H. Liping et al. [22] developed a procedure similar to that described in [21] but replacing the sensors integration platform from a trolley-like vehicle to a road-rail vehicle headframe for the assembly of a overhead lines.

Studies found in the literature show a fundamental limitation in MLS systems; inspection trains cannot operate at assembly phase. These systems do not provide operators with the information they need while performing installation and maintenance tasks. Furthermore, the point clouds captured and used in these studies are postprocessed once MLS systems have finished the inspection. This operation results in a heavy workload for operators and does not allow operators to obtain real time measures on the field. Besides, these MLS use professional grade sensors, instead of commercial ones, resulting in nonportable systems that are less flexible for daily use. Last, the systems described in patents are just proposed solutions with no implementation or evaluation presented.

The review of the literature highlights the need for a system that allows operators to quickly and efficiently check the height and stagger of the contact and messenger wires of a railway infrastructure on the field by means of commercial LiDAR sensor.

## 3. Materials and Methods

**Figure 3:**
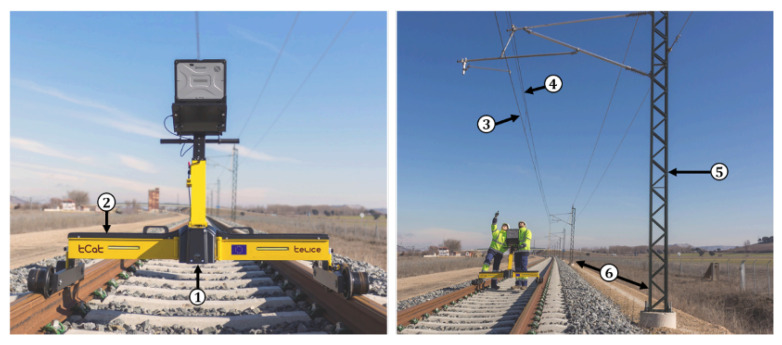
System used to perform the point clouds recording (Telice) in a typical railway environment where its key points; LiDAR (1), trolley (2), contact wire (3), messenger wire (4), mast (5) and mid-span (6) can be distinguished.

This study is focused on the application of a new detection method on point clouds collected by a commercial LiDAR, which does not allow to obtain 360 degree slices and which is placed on a trolley vehicle manually operated over the track. Due to the limited opening angle of the used LiDAR, it is necessary to place the sensor on the trolley with the angle pointing up, which allows a wide enough field of view to detect the key elements of the electrified infrastructure. Figure 3 shows the system used to perform the point clouds recording.

In this case, the navigation system is reduced to a odometer mounted on the trolley which is manually operated over the track. This odometer allows assigning a value to the Z coordinate of each slice captured by the LiDAR. Each of the measurements collected by the sensor include the values needed to calculate the distance to a particular point: the shooting angle, the time needed by the light beam to bounce back to the sensor and the intensity level relative to that beam or Received Signal Strength Indicator (RSSI).

For each measurement of the sensor, the 2D position (X and Y coordinates) of the point in which the beam has bounced can be computed, and then these data can be joined to the Z coordinate provided by the odometer and to the intensity level collected by the sensor as well, resulting in a point formed by values X, Y, Z and intensity.

The resulting point cloud consists of several slices joined with a gap between them directly related with the trolley speed over the track, the update rate from the odometer and the scan speed of the commercial sensor. Gaps between slices of between 20 and 40 centim usually appear when the operator walks at a normal speed (around 4 km/h). The operator can reduce the walk speed in the most interesting points of the infrastructure (typically, key points such as the mid-span and below masts) to achieve a smaller separation between slices and, therefore, to get more data on that section.

Figure 3 shows the system used for collecting the point clouds processed in this study.

Several point clouds from different railway sections with changing features between them have been collected in order to develop a method capable of identifying the contact wires in most of the railway environments.

The techniques mentioned in the related work section are applied to high density point clouds. The characteristics of the point clouds obtained from a commercial LiDAR sensor described above imply that there is a need to develop new contact wire detection methods suitable for this kind of point cloud.

The developed method is subdivided into a series of operations to be carried out on the original point cloud previously divided into sections of approximately one metre in length: selection of points within the region of interest, points clustering, clusters classification, wires building, refinement of the wires, interpolation of straight lines and messenger wires identification. The pseudocode following this operation can be shown in Appendix A.

### 3.1. Selection of Points within the Region of Interest

The first phase of the developed method consists in reducing the number of points of the cloud to be analysed, selecting only those ones within a region of interest.

**Figure 4:**
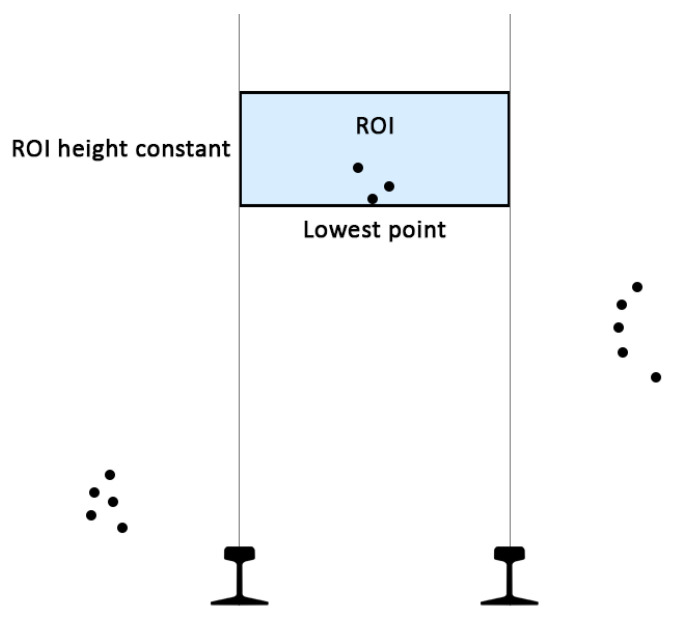
Region of interest computation schema.

As it is shown in Figure 4, the region of interest (ROI) is defined as the area within the lateral limits marked by the track gauge, a bottom limit marked by the first point of the cloud found within the lateral limits and over the sensor and a top limit computed by adding a constant value to the bottom limit.

A dynamic window based on the bottom limit has been chosen due to the changing features between different point clouds that do not allow using a static window.

### 3.2. Points Clustering

The second phase of the developed method aims to carry out a first identification and clusterization of those points which can potentially be part of a key element in the analysed infrastructure, that is, those points that can be part of the contact or messenger wires. For this, a DBSCAN [23] type clusterization is performed on the points within the region of interest of each slice. DBSCAN has been chosen because: (1) it is a simple and quick method; (2) it does not need initial information to work; (3) and it fits our problem because the separation of the clusters can be easily customised.

The DBSCAN algorithm analyses the distance between each point and the clusters already formed. If this distance is less than a predefined threshold epsilon (ϵ), the point is added to that cluster. Otherwise, if the distance is greater than the threshold, the point starts a new cluster by itself.

A value of 28 millim has been selected for the epsilon clustering parameter. This value has been settled by the Telice company. The value is based on the analysis of the results of a test series carried out with the sensor in a real environment, fitting the sensor capabilities and usual distance between the wires (both contact and messenger).

### 3.3. Clusters Classification

The goal of this cluster classification phase is to carry out a first classification as potential points belonging to the infrastructure wires, both contact and messenger ones.

This operation is performed based on the clusters identified in the previous phase of the algorithm. Due to the capability of the LiDAR used and regarding the angle between shots and the usual distance range between the sensor and the wires, it is able to collect between 1 and 3 shots in each wire. However, if two wires are close enough, the sensor uncertainty may cause the DBSCAN algorithm to produce only one cluster. In this case, a cluster representing two wires will have between four and six points.

Given these specifications, the points in a cluster formed of up to 3 points are directly classified as potential points of a contact wire.

**Figure 5:**
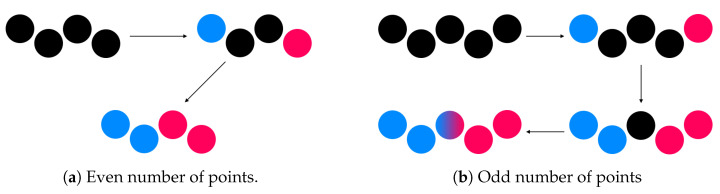
Cluster split process example for clusters that contain between four and six points. (**a**): split process with even number of points; (**b**): split process with odd number of points.

As it can be shown in Figure 5, clusters containing between four and six points are processed in order to split them in two to represent each wire. This split procedure consists of detecting the two farthest points in the cluster, defining two new subclusters initialised with these points and adding the closest point to each subcluster on each iteration. In this way, if the number of points in the cluster is even Figure 5a, the cluster is split into two subclusters representing both wires. However, if the number of points in the cluster is odd Figure 5b, the last unclassified point is doubled and added to both final subclusters. After this procedure, points within both subclusters are included separately in the list of potential points belonging to a wire.

Finally, clusters containing more than six points are discarded and eliminated from the detection algorithm because they represent other types of railway elements other than the wires within the region of interest, typically elements assembled on the posts (cantilever tubes and arms, grips, etc.).

### 3.4. Wires Building

The goal of this phase of the method is to identify which clusters—and therefore which points—make up each overhead wire (both contact and messenger wires) taking as a base the previously classified as potential wires clusters and points.

**Figure 6:**
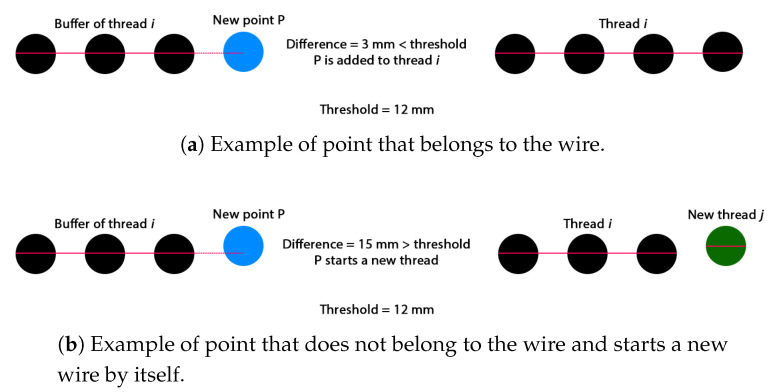
Check performed by the algorithm to label the current point analysed as belonging to that wire or not. (**a**): point that belongs to the wire; (**b**): point that does not belong to the wire.

In order to perform this procedure, the most representative point of each cluster is computed as the intensity-weighted average of all the points in the cluster. Figure 6 shows how the previously obtained point is compared to the growth trend of each wire and, if the point is between a predefined margins (Figure 6a), all points in the cluster are labelled as belonging to that wire. If, on the other hand, the representative point of the cluster is not within the growth margins of any of the wires (Figure 6b), a new wire will be created with the points of that cluster.

This growth trend is implemented as a buffer. A customisable parameter *N* defines the number of clusters within a wire that will be taken into account to compute the trend. The adjustment of this parameter will depend on each specific cloud and the distance between slices. The closer the slices are, the higher the parameter N can be. If the last *N* clusters added to a wire show that it grows in a stable way (without variations in height), the next cluster added to the wire must maintain this trend and not assume a deviation in the height of the wire. A buffer has been chosen to implement the growth trend because the variability in the consistency of the point clouds did not allow one to establish a growth trend that only takes into account the last clusters added to each wire.

### 3.5. Refinement of the Wires

The next phase aims to eliminate false positives in the wires detected in the previous phase.

A procedure was implemented for the candidate wires identified so far to be analysed looking for those ones that do not have the expected length. This expected length is determined based on the longest detected wire (reference wire), typically with the same length as the analysed section.

The use of a filter based on the length of the wires has been chosen because the results obtained by applying a filter based on the number of points in the wires were not consistent due to the difference in the density of points between different point clouds and even between different sections of the same point cloud.

### 3.6. Interpolation of Straight Lines

Due to the variable morphology of the points that form each of the wires, a more clear representation of each of the wires is necessary. This representation is achieved by interpolating the straight line that best fits each set of points that form the wires. In this way, each wire—both contact and messenger wires—is represented by a straight line, allowing making measurements to the straight line to be a unique representation of the wire.

The interpolation is carried out by the application of a Principal Component Analysis (PCA) procedure [24]. This is a traditional algorithm used to set the straight line that best fits to a set of points. The algorithm defines a line that minimises the average squared distance from the points to the line. Once the straight line is available, a measurement of the key parameters can be performed over it, that is, compute its height with respect to the track’s rail level and its stagger to the track axis.

### 3.7. Messenger Wires Identification

The last phase of the method consists in identifying and classifying those points that can be part of messenger wires rather than contact wires. For this, the height and stagger of each wire is analysed and classified as a messenger wire if it is in a range specified by the operator above the contact wire and with a stagger similar to it.

## 4. Results

The detection method has been tested with two point clouds corresponding to a test bench and a real railway environment. Measures of the contact wires were taken in both scenarios using the new method and the tool commonly used, that is, the laser rangefinder, in order to compare the performance of both procedures.

### 4.1. Scenarios and Datasets

The first scenario was a section of a real railway infrastructure, specifically a tunnel.

**Figure 7:**
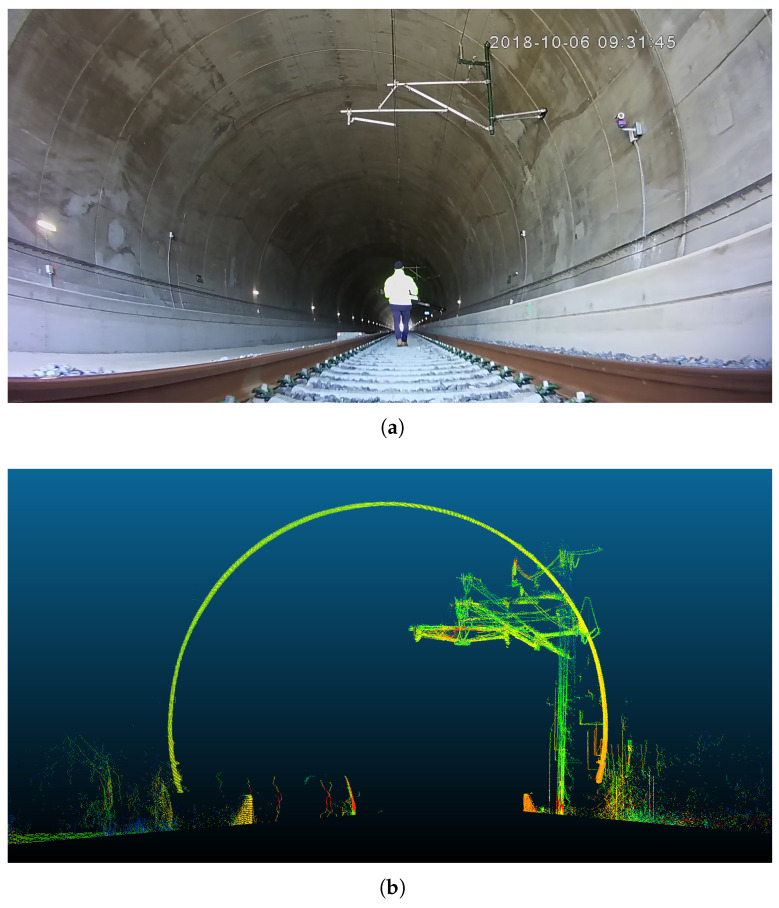
Real tunnel railway environment in which real environment data was collected. (**a**): picture from real environment; (**b**): point cloud recorded in the real environment.

A picture from the real environment is shown in Figure 7a and a frontal view of the point cloud recorded in that environment is shown in Figure 7b.

The points density for each metre of the point clouds is a critical feature for the proper working of the proposed method. The more density of the points, the better performance of the proposed method. In this case, the operators walked on the section of the track at a moderate speed and in a single direction, which results in a point cloud with a density of approximately 2500 points per metre.

**Figure 8:**
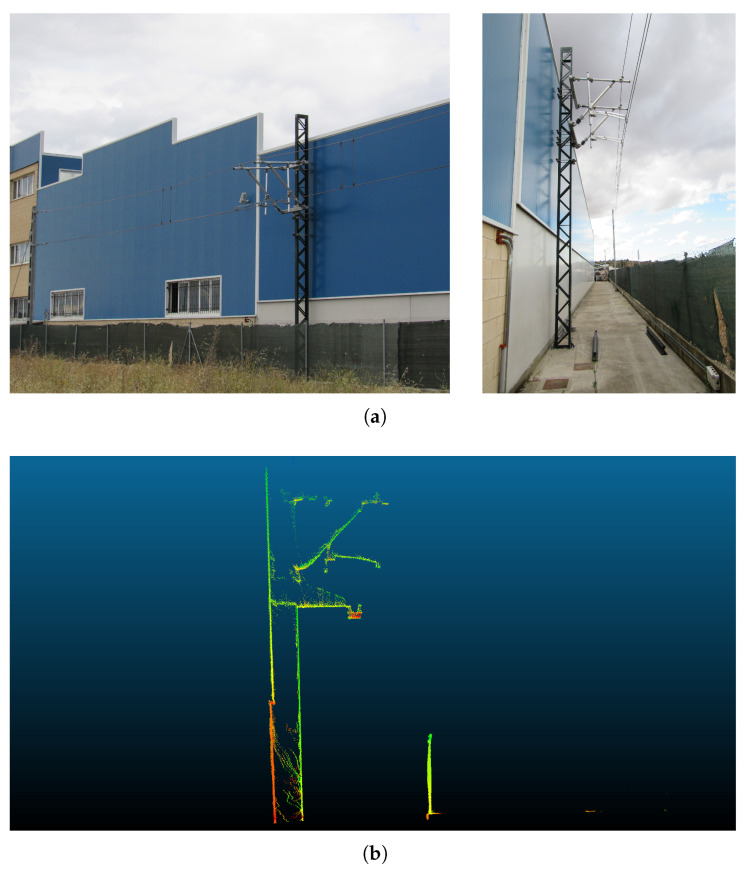
Test bench environment in which test bench data was collected. (**a**): picture from test bench environment; (**b**): point cloud recorded in test bench environment.

The second scenario corresponds to a test bench that reconstructs a real railway environment. Pictures from the test bench environment are shown in Figure 8a,b shows a frontal view of the point cloud recorded in that environment.

In this case, the point cloud was collected by successive forward and backward passes in the test bench environment, which results in a higher point density per metre (approximately 231,000 points).

### 4.2. Evaluation

To carry out an objective evaluation of the developed method, both height and stagger measures obtained with a Leica DISTOTM D510 laser rangefinder like the one shown in Figure 1 are compared with those obtained through the proposed method. Comparison with the laser rangefinder has been selected because it is commonly used for measuring, in addition to being a direct comparison between a manual method and the new automatic detection and measurement method. Laser rangefinder measurement consists of placing the ruler itself over the rails. Then the operator must point the laser to the wire that needs to be measured and note both height measurement provided by the laser and stagger measurement provided by the ruler over the rails.

The measurements with the laser rangefinder were made at the points of interest of the track for operators, typically under the posts and in the mid-span. Said measurement points were noted and subsequent measurements were made at the same points using the proposed method.

### 4.3. Measurements

**Figure 9:**
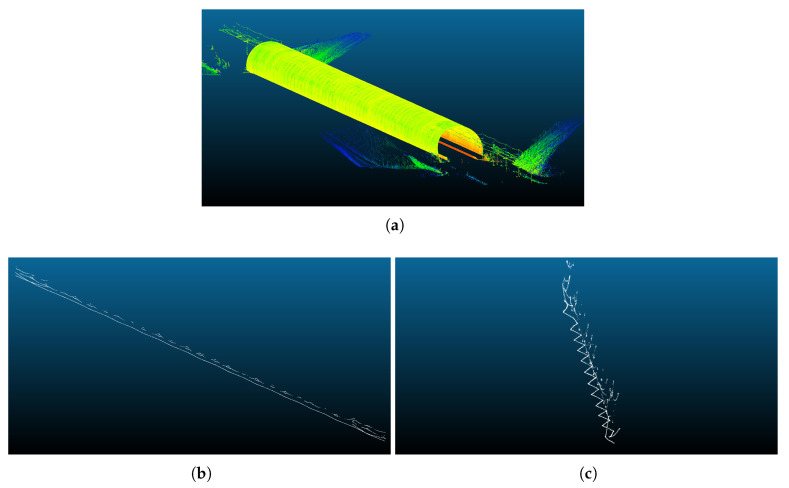
Point cloud recorded in a real tunnel railway environment and the points classified by the method as belonging to a wire in this point cloud. (**a**): a perspective of the point cloud recorded in the real environment; (**b**): points classified as contact or messenger wires by the new method; (**c**): a different perspective of the classified points.

From the whole point cloud shown in Figure 9a, the points classified by the new method as contact or messenger wires are shown in Figure 9b. Figure 9c shows a different perspective of the classified points in order to observe how the wires weave from side to side along the tunnel.

**Figure 10:**
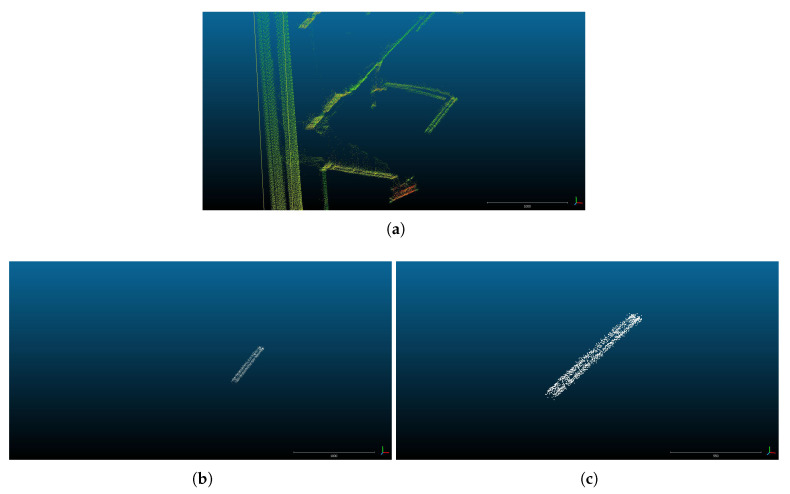
Point cloud recorded in a test bench environment and the points classified by the method as belonging to a wire in this point cloud. (**a**): an amplified perspective centred in the overhead contact line of the test bench environment point cloud; (**b**): points classified as contact wire by the new method; (**c**): a different perspective of the classified points.

Figure 10a shows an amplified perspective centred in the overhead contact line of the test bench environment point cloud. In Figure 10b, only the points classified by the new method as contact wires are shown. Figure 10c shows an amplified perspective of the previous one.

**Figure 11:**
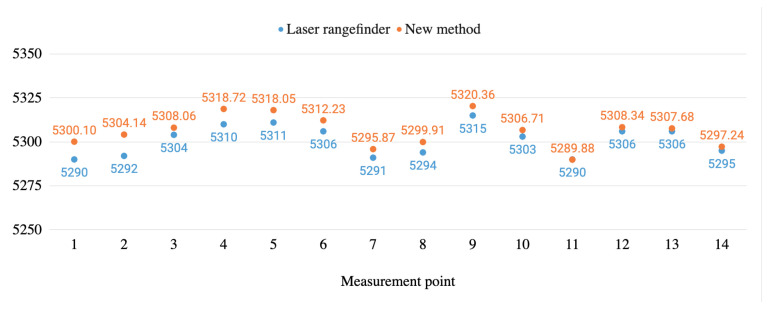
Height measurements’ comparison between the new method and the laser rangefinder in the real infrastructure environment.

**Figure 12:**
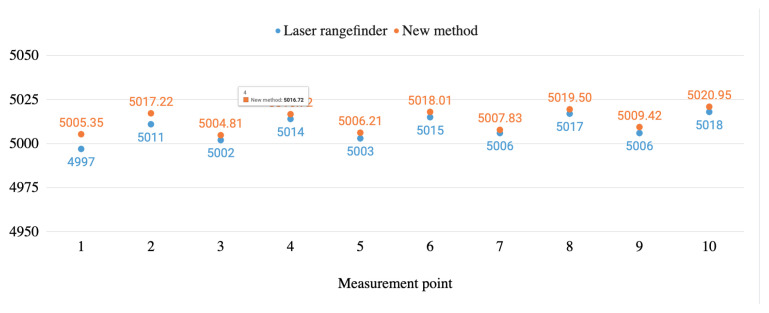
Height measurements’ comparison between the new method and the laser rangefinder in the test bench environment.

Figure 11 and Figure 12 graphically show the height measurement comparisons in millim between the laser rangefinder and the proposed method carried out both in a real infrastructure and in a test bench.

As it is shown in these figures, the measurements from the laser rangefinder are always below the ones obtained by using the method proposed. The difference between the diameter of the beam of light of the rangefinder and the LiDAR can explain this situation. For both devices, when they point to elements in the range of 4.5–6 m, the first one works with a beam which is 3 mm wide while for the second one it is around 37 mm wide (source: devices’ manufacturers), while the wire is typically 14 mm wide. When working with the laser rangefinder, the operator centres the shot in the wire and the measure is not taken until the whole red point hits the wire, assuring that it is placed in its lower part. When working with the LiDAR, more than one shot is recorded. Taking into account the width of the wire, the width of the beam of light and the shot frequency, unlike the laser rangefinder, more than one point is obtained when the LiDAR beam impacts the whole wire. The measure taken for every shot can correspond to the lower part or to the sides of the wire. The proposed method interpolates using these points generating measures that will always be slightly above the ones obtained with the laser rangefinder. Nonetheless, the new method saves time and possible human errors.

**Figure 13:**
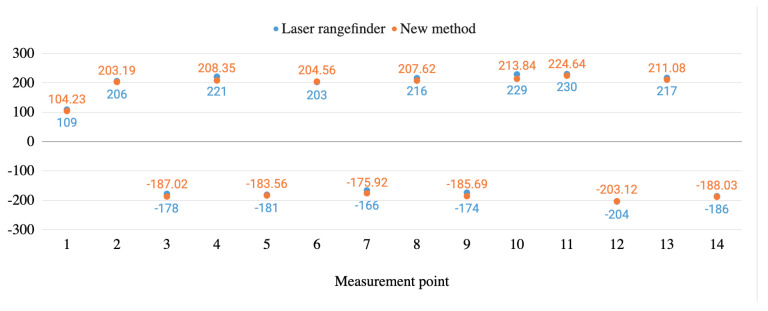
Stagger measurements’ comparison between the new method and the laser rangefinder in the real infrastructure environment.

**Figure 14:**
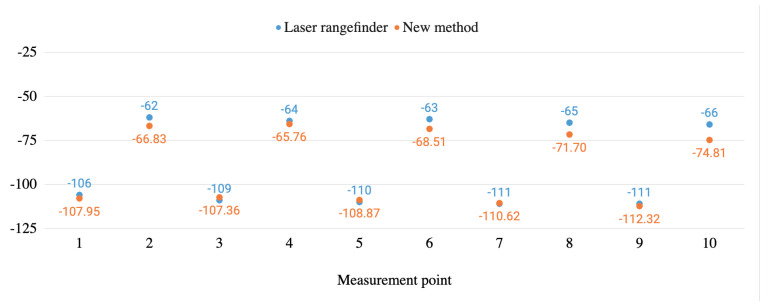
Stagger measurements’ comparison between the new method and the laser rangefinder in the test bench environment.

Figure 13 and Figure 14 graphically show the stagger measurement comparisons between the laser rangefinder and the proposed method carried out both in a real infrastructure and in a test bench.

Table 1 shows height and stagger measurement differences in the measurement points between both point clouds, as well as the average and typical deviation of those differences.

## 5. Discussion

**Figure 15:**
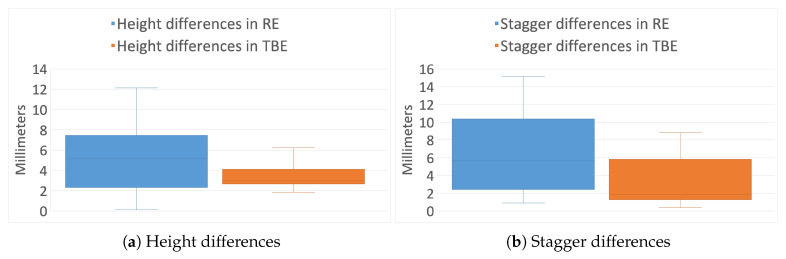
Height and stagger differences’ distribution in real and test bench environments.

Results show a height average difference of 5.32 mm and 6.62 mm for the stagger in the real infrastructure environment, while these differences are reduced to 3.70 mm and 3.40 mm respectively in the test bench environment. Figure 15a graphically shows the distribution of the differences in height, and Figure 15b shows the differences in stagger. In both figures, the first box corresponds to the real infrastructure environment, and the second box represents the differences in the test bench environment.

A smaller difference between measurements is shown in the data from the test bench environment, both in height and stagger. Taking into account that the points were obtained in each environment using the same equipment, but with the only difference of the number of points taken, we can conclude that the quality of the point cloud is relevant in the final results. That is, a higher points’ density is desirable for obtaining more accurate measurements from the proposed algorithm.

Regarding the procedure used to measure, the comparison made between the algorithm and the laser rangefinder has been carried out because the latter is commonly used for it. However, height measurements with the laser rangefinder are made by an operator when he visually detects that the laser is pointing towards the contact wire. The operator must manually place the laser in the position where he detects the laser spot in the wire, and at this point the height measurement (provided by the laser distance metre) is taken. At this moment, the operator manually notes the distance of the laser to the centre of the graduated ruler placed on the track, thus obtaining the measurement of the contact wire stagger. This full manual procedure is prone to multiple human errors, such as not spotting the wire properly or making mistakes when writing down the data. The proposed algorithm provides an automated procedure to measure both height and stagger with no human intervention needed, which improves the procedure in two ways. On the one hand, the whole process saves time, and on the other hand, it is much less prone to human errors.

Last but not least, using this method, not only the contact wire measurements are obtained, but the rest of the points inside the range of the laser, including the rest of the elements of the rail infrastructure, such as, masts or platforms. The same proposed procedure can be adapted offline in order to measure other elements of the cloud obtained.

## 6. Conclusions and Future Work

A new method for the automatic extraction of power cables location in railways using surface LiDAR systems has been presented. Following the initial goals proposed, the method can be applied both in assembly and maintenance phases, offering real time measures on the field that are essential for this kind of operation. The system that integrates the new method works with commercial LiDAR sensors, as initially required.

Unlike MLS mounted on inspection trains, this system is portable by two men, which implies much lower operation costs. The cost of these trains is in the order of millions of euros. If the comparison is made with the laser rangefinder, then some parameters have to be established. For instance, to inspect a 10 km track, the process will take one working day with the proposed system and four working days with the laser rangefinder. The approximate cost in the first case, including salaries, equipment rental, subsistence, etc., would be around 650 € and 1600 € in the second case.

Furthermore, the method described and the final system where it is integrated in, assure a much lower human error rate thanks to the higher level of automation.

An algorithm refinement is proposed as future work. This refinement can improve the algorithm in order to improve its performance with other kinds of railway infrastructures, what would allow its generalisation to different point clouds. Likewise, the detection and characterisation of other elements of the infrastructure, such as masts, platforms, etc. is presented as a very interesting future research work.

## Figures and Tables

**Table 1:** Differences between compared methods.

Differences in RE			Differences in TBE		
Measurement Point	Height	tagger	Measurement Point	Height	Stagger
1	10.10 mm	4.78 mm	1	8.35 mm	1.94 mm
2	12.14 mm	2.82 mm	2	6.22 mm	4.83 mm
3	4.06 mm	9.02 mm	3	2.81 mm	1.64 mm
4	8.72 mm	12.65 mm	4	2.72 mm	1.76 mm
5	7.05 mm	2.56 mm	5	3.21 mm	1.13 mm
6	6.23 mm	1.56 mm	6	3.01 mm	5.51 mm
7	4.87 mm	9.92 mm	7	1.83 mm	0.38 mm
8	5.91 mm	8.38 mm	8	2.50 mm	6.70 mm
9	5.36 mm	11.69 mm	9	3.42 mm	1.32 mm
10	3.71 mm	15.16 mm	10	2.95 mm	8.81 mm
11	0.12 mm	5.36 mm
12	2.34 mm	0.88 mm
13	1.68 mm	5.92 mm
14	2.24 mm	2.03 mm			
$\bar{X}$	5.32 mm	6.62 mm	$\bar{X}$	3.70 mm	3.40 mm
œ	3.38 mm	4.56 mm	œ	2.00 mm	2.85 mm

## Abbreviations

   The following abbreviations are used in this manuscript:

RE	Real environment
TBE	Test bench environment

## Appendix A. New Method Pseudocode

**Algorithm 1** Algorithm for building contact wires		
1:	*roiPoints* ← extractPointsFromROI()	▹ First stage

2:	**for all** *slice* ∈ *roiPoints* **do**	▹ Second stage
3:	*clusteredPoints* ← DBSCAN(*slice*)	
4:	**end for**	

5:	*finalClusters* ← *NULL*	▹ Third stage
6:	**for all** *cluster* ∈ *clusteredPoints* **do**	
7:	**if** *cluster.Length* > 6 **then**	
8:	*continue*	
9:	**else if** *cluster.Length* > 3 **then**	
10:	*splittedCluster* ← SPLIT(*cluster*)	
11:	*finalClusters.Add*(*splittedCluster*)	
12:	**else**	
13:	*finalClusters.Add*(*cluster*)	
14:	**end if**	
15:	**end for**	

16:	*listO fWires* ← *NULL*	▹ Fourth stage
17:	**for all** *cluster* ∈ *finalClusters* **do**	
18:	*closestWire* ← getGlosestWireFromList(*cluster*)	
19:	**if** *closestwire.Distance* < ϵ **then**	
20:	*closestWire.Add*(*cluster*)	
21:	**else**	
22:	*newWire* ← *cluster*	
23:	*listO fWires.Add*(*newWire*)	
24:	**end if**	
25:	**end for**	

26:	*longerWire* ← getGlongerWire(*listO fWires*)	▹ Fifth stage
27:	**for all** *wire* ∈ *listO fWires* **do**	
28:	**if** *wire.Length*< *longerWire.Length* * 0, 5 **then**	
29:	*listO fWires.Remove*(*wire*)	
30:	**end if**	
31:	**end for**	

32:	**for all** *wire* ∈ *listO fWires* **do**	▹ Sixth stage
33:	computeBestFirStraitht(*wire*)	
34:	measureHeithtAndStagger(*wire*)	
35:	**end for**	

36:	**for all** *wire* ∈ *listO fWires* **do**	▹ Seventh stage
37:	**for all** *potentialSustainer* ∈ *listO fWires* **do**	
38:	**if** isSustaniner(*potencialSustainer,wire*) **then**	
39:	*wire.AddSustainer*(*potencialSustainer)*	
40	**end if**	
41:	**end for**	
42:	**end for**	
